# Supplementary material for: A proteomics analysis of 5xFAD mouse brain regions reveals the lysosome-associated protein Arl8b as a candidate biomarker for Alzheimer’s disease
Source: Genome Med. 2023 Jul 20;15:50. doi: 10.1186/s13073-023-01206-2 (PMC10357615; doi:10.1186/s13073-023-01206-2)
Supplement: Supplementary file 1 — Additional file 1: Table S1. Commercial antibodies used in this study. Table S2. Characteristics of AD and HD patients and corresponding controls. Table S3. Spearman correlation analysis of Arl8b protein level measurements and AD biomarker levels. [file 13073_2023_1206_MOESM1_ESM.docx]

| \| **Name** \| **Supplier** \| **Cat. No. / RRID** \| **Use in this study** \| \| --- \| --- \| --- \| --- \| \| Anti-Arl8b \| Proteintech \| 13049-1-AP / AB_2059000 \| IB, IS \| \| Anti-Arl8a/b \| LSBio \| LS-C379507 \| IB \| \| Anti-β-Amyloid, 1-16 (clone 6E10) \| BioLegend \| 803002 / AB_2564654 \| IB \| \| Anti-Lamp1 \| Santa Cruz Biotechnology \| sc-19992 / AB_2134495 \| IB, IS \| \| Anti-Calnexin \| Stressgen \| SPA-860 \| IB \| \| Anti-VDAC \| Cell Signaling Technology \| 4661 / AB_10557420 \| IB \| \| Anti-Golgin97 \| Cell Signaling Technology \| 13192 / AB_2798144 \| IB \| \| Anti-Flotillin \| Cell Signaling Technology \| 3253 / AB_2106734 \| IB \| \| Anti-NDUFB3 \| Abcam \| Ab202585 / AB_2890186 \| IB \| \| Anti-α-Tubulin \| Sigma Aldrich \| T6074 / AB_477582 \| IB \| \| Anti-α-Tubulin \| Sigma-Aldrich \| SAB3501072 \| IB \| \| Anti-Presenilin-1, NT, clone 2Q127 \| United States Biological \| P6300-35C \| IB \| \| Anti-Amyloid β (N) (clone 82E1) \| Immuno-Biological Laboratories \| JP10326 / AB_2341281 \| IS \| \| Anti-rabbit IgG peroxidase \| Sigma-Aldrich \| A0545 / AB_257896 \| IB \| \| Anti-mouse IgG peroxidase \| Sigma-Aldrich \| A0168 / AB_257867 \| IB \| \| Alexa Fluor 594 anti-β-Amyloid, 1-16 (clone 6E10) \| Biolegend \| 803002 / AB_2564654 \| IS \| \| Goat anti-Rat IgG (H+L) Cross-Adsorbed Sec. Ab, Alexa Fluor 594 \| ThermoFisher Scientific \| A-11007 / AB_10561522 \| IS \| \| Goat anti-Mouse IgG (H+L) Cross-Adsorbed Sec. Ab, Alexa Fluor 594 \| ThermoFisher Scientific \| A-11005 / AB_2534073 \| IS \| \| Goat anti-Rabbit IgG (H+L) Cross-Adsorbed Sec. Ab, Alexa Fluor 647 \| ThermoFisher Scientific \| A-21244 / AB_2535812 \| IS \|  \| IS, immunostaining; IB, immunoblotting \| \| --- \| \|  \| |
| --- | --- | --- | --- | --- | --- | --- | --- | --- | --- | --- | --- | --- | --- | --- | --- | --- | --- | --- | --- | --- | --- | --- | --- | --- | --- | --- | --- | --- | --- | --- | --- | --- | --- | --- | --- | --- | --- | --- | --- | --- | --- | --- | --- | --- | --- | --- | --- | --- | --- | --- | --- | --- | --- | --- | --- | --- | --- | --- | --- | --- | --- | --- | --- | --- | --- | --- | --- | --- | --- | --- | --- | --- | --- | --- | --- | --- | --- | --- | --- | --- | --- | --- |

**Table S1: Commercial antibodies used in this study**

| **CSF of AD patients and controls** |  |  |  |  |  |  |  |  |  |
| --- | --- | --- | --- | --- | --- | --- | --- | --- | --- |

| **Characteristics** | **Controls (n = 44)** | **AD (n =38)** |
| --- | --- | --- |
| *Demographic data* |  |  |
| Age in years at LP (range) | 66.16 (47-80) | 75,05 (60-87) |
| Female sex, n (%) | 23 (52.27%) | 20 (52.6%) |
| *Clinical data* |  |  |
| MMSE score at LP, mean (range), n | 29.03 (26-30), n=32 | 23.1 (12-30), n=38 |
| *Biomarker data* |  |  |
| CSF t-tau pg/ml (range) | 268.59 (163-399) | 732.53 (405-1505) |
| CSF p-tau pg/ml (range) | 38.5 (18-57.2) | 111.04 (72-279.2) |
| CSF Aß(1-42) pg/ml (range) | 1170 (724-2110) | 484,5 (229-649) |
| CSF Aß(1-40) pg/ml (range) | 11693.64 (7491-18279) | 10996.82 (4615-18924) |
| CSF Aß(1-42)/Aß(1-40) (range) | 0.0998 (0.059-0.127) | 0,0448 (0.025-0.054) |

|  |  |  |  |  |  |  |  |  |  |
| --- | --- | --- | --- | --- | --- | --- | --- | --- | --- |
| **CSF of HD patients and controls** |  |  |  |  |  |  |  |  |  |

| **Characteristics** | **Controls (n = 10)** | **HD (n = 10)** |
| --- | --- | --- |
| *Demographic data* |  |  |
| Age in years at LP (range) | 36.6 (27-60) | 46 (25 – 59) |
| Female sex, n (%) | 7 (70%) | 5 (50%) |

LP, lumbar puncture

**Brain samples of AD patients and controls, cohort 1, used to generate Figures 6a and b**

| **Characteristics** | **Controls (n=10)** | **AD (n=10)** |
| --- | --- | --- |
| *Demographic data* |  |  |
| Age at death (range) | 69.7 (59-75) | 72.2 (63-80) |
| Female sex, n (%) | 3 (30%) | 4 (40%) |
| *Neuropathological data* |  |  |
| Braak stage (range), n | 0.44 (0-1), n=9 | 5.71 (5-6), n=7 |

**Brain samples of AD patients and controls, cohort 2, used to generate Figures S14a and b**

| **Characteristics** | **Controls (n=10)** | **AD (n=10)** |
| --- | --- | --- |
| *Demographic data* |  |  |
| Age at death (range) | 69.7 (59-75) | 73 (63-80) |
| Female sex, n (%) | 3 (30%) | 4 (40%) |
| *Neuropathological data* |  |  |
| Braak stage (range), n | 0.44 (0-1), n=9 | 5.43 (4-6), n=7 |

**Table S2: Characteristics of AD and HD patients and corresponding controls**

| **Biomarker** | **Spearman correlation coefficient *r_S_*** | **p value** |
| --- | --- | --- |
| Aβ(1-42) | -0.316 | 0.0038 |
| Aβ(1-40) | 0.129 | 0.2466 |
| Aβ42/Aβ40 | -0.405 | 0.0002 |
| t-tau | 0.366 | 0.0007 |
| p-tau | 0.415 | 0.0001 |

| **Table S3: Spearman correlation analysis of Arl8b protein level measurements and AD biomarker levels.** The statistical significance of the association was measured with a two-tailed t-test. T-tau, total tau. P-tau, p181 phosphorylated tau. |  |
| --- | --- |
