# Supplementary material for: A proteomics analysis of 5xFAD mouse brain regions reveals the lysosome-associated protein Arl8b as a candidate biomarker for Alzheimer’s disease
Source: Genome Med. 2023 Jul 20;15:50. doi: 10.1186/s13073-023-01206-2 (PMC10357615; doi:10.1186/s13073-023-01206-2)
Supplement: Supplementary file 2 — Additional file 2: Fig. S1. Aβ peptide levels and APP and PSEN1 expression in hippocampus and cortex of 5xFAD mice. Fig. S2. Analysis of Aβ aggregate formation using membrane filter assays and sucrose gradient centrifugations. Fig. S3. Analysis of wild-type expression profiles to assess whether the protein abundance changes detected in 5xFAD brains are more frequent among highly expressed mouse proteins. Fig. S4. Functional analysis of dysregulated proteins defined with a pairwise model in brains of 5xFAD mice. Fig. S5. Enrichment analysis of cell-type-specific marker proteins among dysregulated proteins in brains of 5xFAD mice. Fig. S6. IPA and gene ontology enrichment analysis of differentially expressed proteins defined with the full model in cortical and hippocampal tissues of 5xFAD mice. Fig. S7. Ingenuity pathway analysis of Aβ-correlated and anticorrelated DEPs defined by the pairwise model in brains of 5xFAD mice. Fig. S8. Numbers of pairwise common DEPs in the mouse datasets and datasets from human studies. Fig. S9. Strategy to define mouse protein signatures that are concordantly altered also in AD patient brains. Fig. S10. Investigation of the overlap of DEPs in brains of 5xFAD mice with DEPs in asymptomatic AD brains. Fig. S11. Analysis of the correlation in protein effect sizes between 5xFAD mouse and AD patient brains for proteins present in all studies. Fig. S12. Selection of the neuronal lysosome-associated protein Arl8b by step-by-step data filtering. Fig. S13. Immunofluorescence analysis of 5xFAD brain slices. Fig. S14. Analysis of Arl8b protein aggregates using human brain homogenates derived from AD patients and control individuals. [file 13073_2023_1206_MOESM2_ESM.pdf]

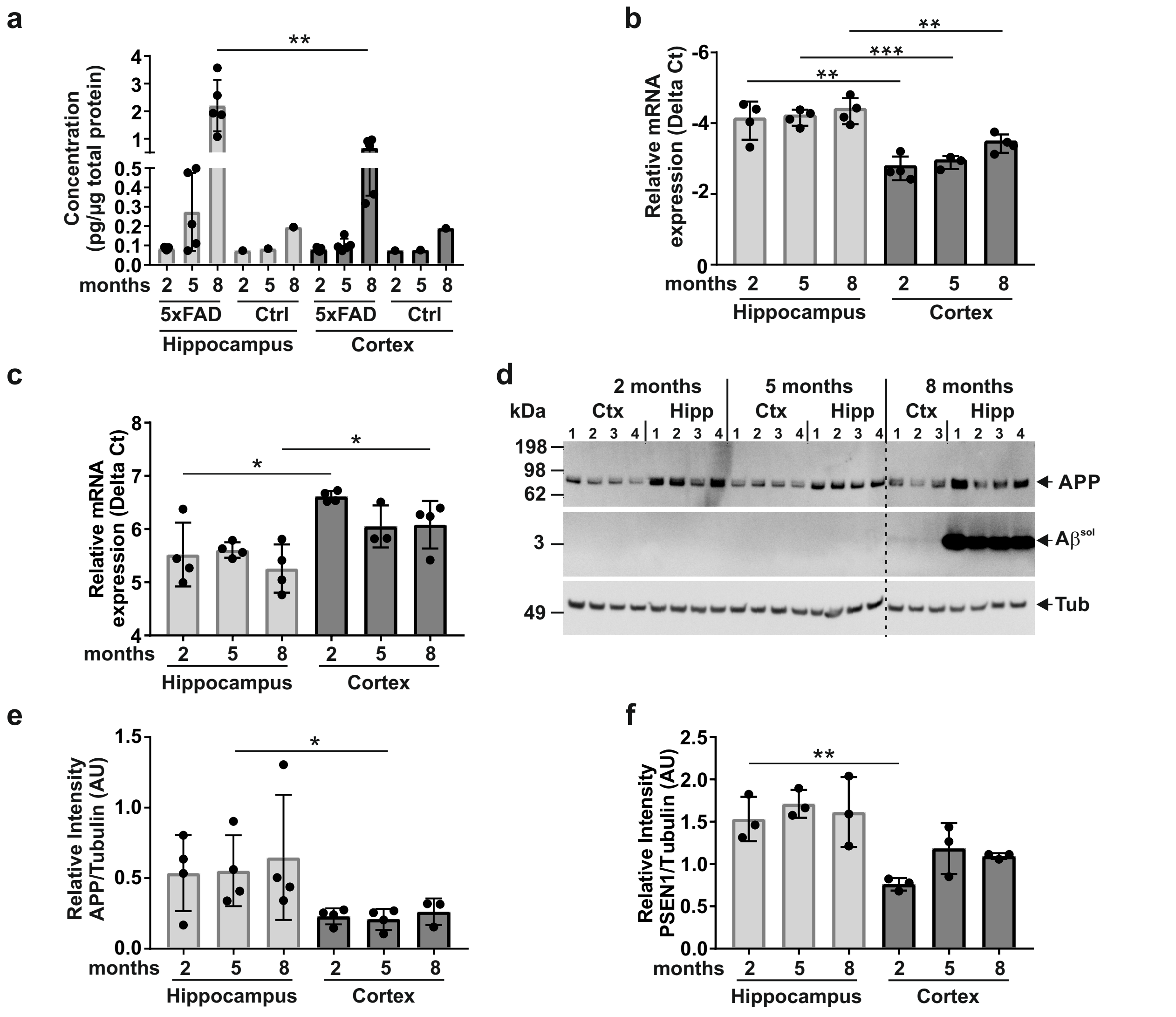

Figure S1. Aβ peptide levels and APP and PSEN1 expression in hippocampus and cortex of 5xFAD mice.

(a) Hippocampal and cortical brain extracts of aged AD mice (n=5) were used for Aβ40 peptide ELISA. As a control, a pool of wt mouse brain extracts per age and tissue was tested as described in **Figure 1a**. Data represent the mean ± SD of five biological replicates of tg mice per age and tissue. The statistical significance was assessed between hippocampal and cortical tissues of the same age using an unpaired, two-tailed t test (**, p = 0.0077). **(b)** Real Time PCR for quantification of human APP transcript levels in hippocampus and cortex of 2-, 5- and 8-month-old 5xFAD mice. Per age and tissue reverse transcribed cDNAs of four different mice were analyzed using a human APP specific TaqMan gene expression assay. Data were normalized to endogenous mouse EIF-4H and represent the mean ± SD. Statistical analysis was performed using an unpaired two-tailed t test comparing transcript levels of hippocampal and cortical tissues of the same age (2 months Hipp/Ctx: p = 0.0054, 5 months Hipp/Ctx: p = 0.0005, 8 months Hipp/Ctx: p = 0.0065). **(c)** Real time PCR for quantification of human PSEN1 transcript levels in hippocampus and cortex of 2-, 5- and 8-month-old 5xFAD mice. Per age and tissue reverse transcribed cDNAs of three to four different mice were analyzed using a human PSEN1 specific TaqMan gene expression assay. Data were normalized to endogenous mouse GAPDH and represent the mean ± SD. Statistical analysis was performed using an unpaired two-tailed t test comparing transcript levels of hippocampal and cortical tissues of the same age (2 months Hipp/Ctx: p = 0.0115, 8 months Hipp/Ctx: p = 0.041). **(d)** Hippocampal (Hipp) and cortical (Ctx) brain extracts derived from four different 5xFAD mice were analyzed by immunoblotting using 6E10 and anti–α-tubulin (Tub) antibodies (#T6074). Antibody 6E10 recognizes human APP and soluble Aβ ($A\beta^{sol}$). The dashed line indicates that the samples of 8 months old mice run on a separate gel under the same experimental conditions. **(e)** APP and tubulin immunoblot intensities **(d)** were quantified using Image J. Relative intensity values (mean ± SD) are shown for hippocampal and cortical tissues of 2-, 5- and 8-month-old 5xFAD mice (n=4). Statistical significance was assessed with an unpaired, two-tailed t test (*, p = 0.039). **(f)** Presenilin-1 and alpha-Tubulin (#SAB3501072) expression were quantified by immunobloting using the iBright Analysis Software (Thermo Fisher Scientific). Relative intensity values (mean ± SD) are shown for hippocampal and cortical tissue of 2-, 5- and 8-month-old 5xFAD mice (n=3). Statistical significance was assessed with an unpaired, two-tailed t test (**, p = 0.081).

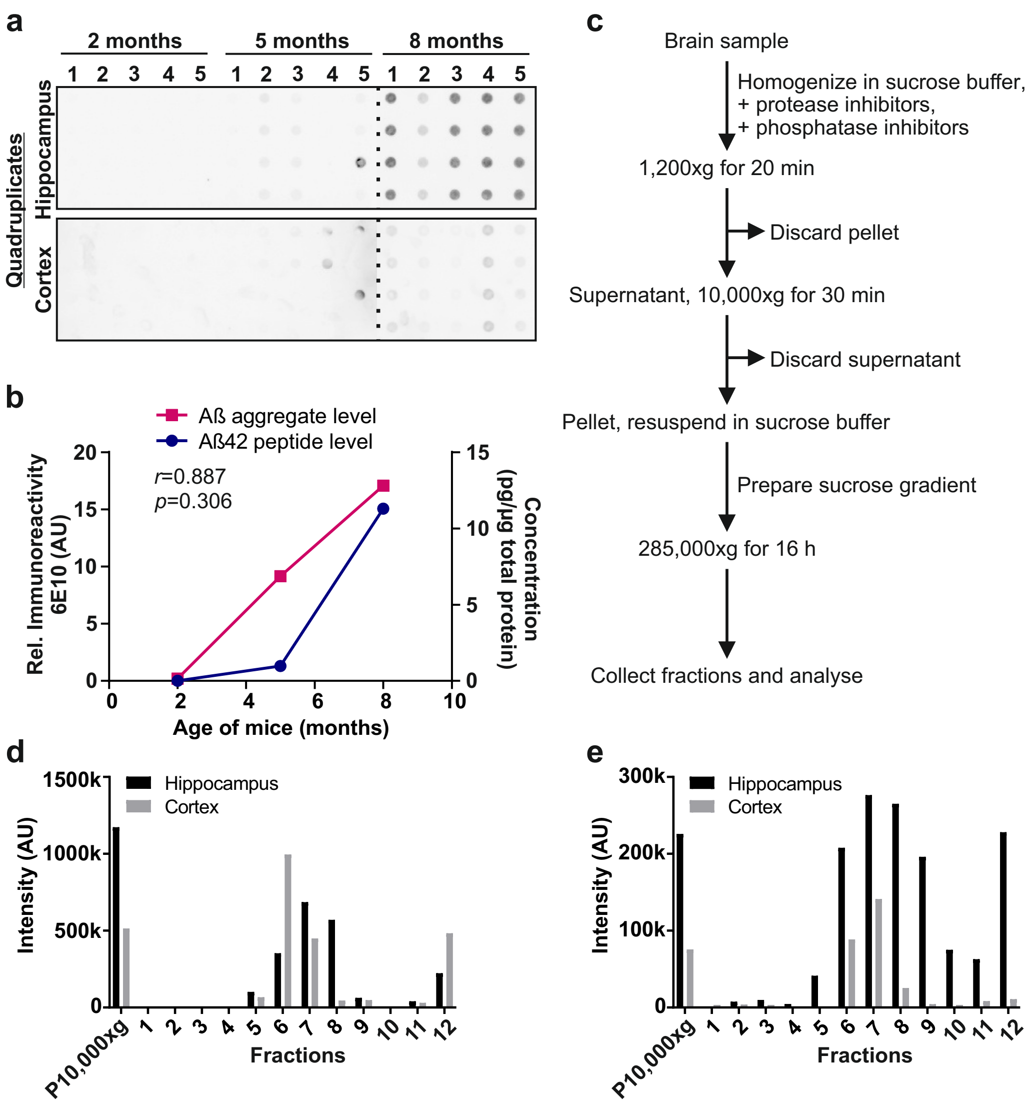

Figure S2. Analysis of Aβ aggregate formation using membrane filter assays (MFA) and sucrose gradient centrifugations.
(a) Membrane filter assay (MFA) of hippocampal and cortical brain extracts of 2-, 5- and 8-month-old 5xFAD mice. Per age, brain extracts of 5 different mice in quadruplicates were tested. Hippocampal and cortical extracts were analyzed on separate filter membranes. For immunoblotting the antibody 6E10 was used. Both filter membranes were exposed for 1 min. The dashed line indicates where the filter was cut. **(b)** Pearson correlation analysis of Aβ42 peptide levels determined by ELISA (blue line, right axis) and Aβ aggregate levels determined by membrane filter assay (purple line, left axis) in cortical tissue samples. The statistical significance of the association between the Aβ42 peptide levels and Aβ aggregate levels was measured with a two-tailed t-test (not significant, p = 0.306). The correlation coefficient *r* and the p-value are given in the upper left corner of the diagram. **(c)** Schematic representation of membrane fraction purification from mouse hippocampal and cortical brain samples using sucrose gradient centrifugation. **(d, e)** Quantification of soluble APP **(d)** and insoluble Aβ aggregates **(e)** in gel pockets of immunoblots in Figure 1g was performed using the iBright Analysis Software (Thermo Fisher Scientific). Immunoblots from hippocampal and cortical fractions for one and the same antibody were prepared under the same conditions.

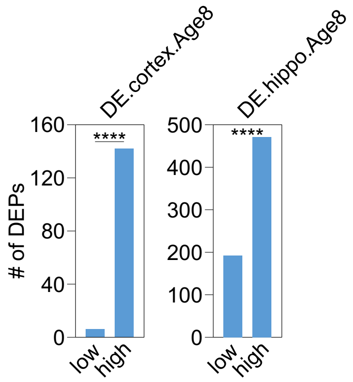

Figure S3. Analysis of wild-type expression profiles to assess whether the protein abundance changes detected in 5xFAD brains are more frequent among highly expressed mouse proteins.
For the differentially altered proteins from DE.Cortex.Age8 and DE.hippo.Age8, a median split was performed and groups of low, medium and high wild-type protein expression were formed, of which the medium group was omitted. The statistical significance was measured with a right-tailed Fisher's exact test (****, $p < 0.0001$). The analysis is based on mean values of measured intensities from five biological replicates of tg mice per age and tissue (n=5).

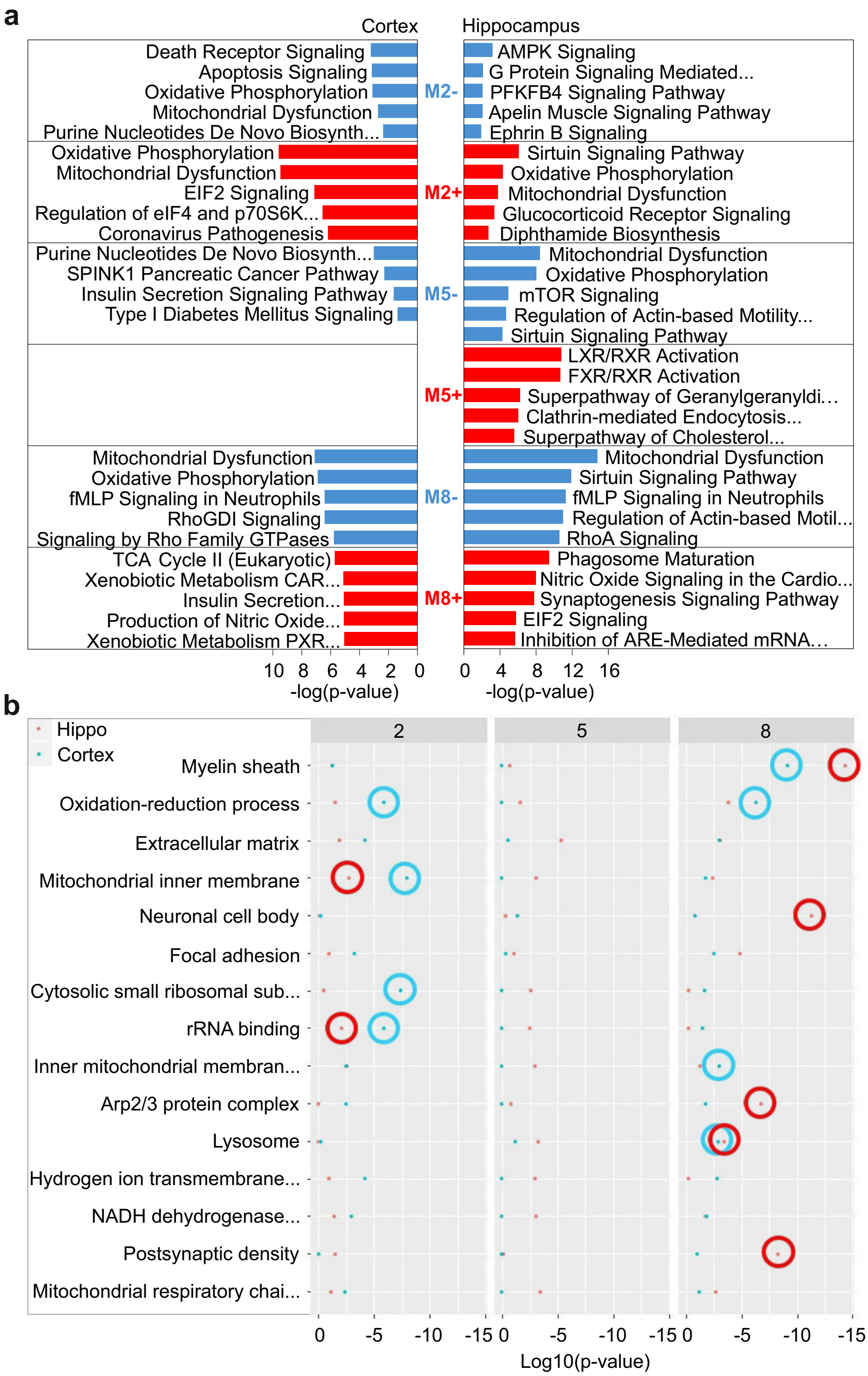

Figure S4. Functional analysis of dysregulated proteins defined with a pairwise model in brains of 5xFAD mice.

(a) Ingenuity pathway analysis (IPA) to assess altered cellular processes of dysregulated proteins in hippocampal and cortical tissues; blue indicates downregulated pathways ("-"), red designates upregulated pathways ("+") at 2, 5 and 8 months (M2, M5, M8). The statistical significance was measured with a right-tailed Fisher's exact test to calculate the p-values, adjusted by the Benjamini-Hochberg multiple testing correction. **(b)** Temporal gene ontology (GO) enrichment analysis of DEPs of 5xFAD mouse brains from both hippocampus and cortex at months 2, 5 and 8. Statistical significance was determined using a right-tailed Fisher's exact test to calculate p-values; they are shown as log10 p-values. Highly significant pathways of interest in hippocampus are marked with red circles, in cortex with blue circles. All analyzes are based on mean values of measured intensities from five biological replicates of tg mice per age and tissue (n=5).

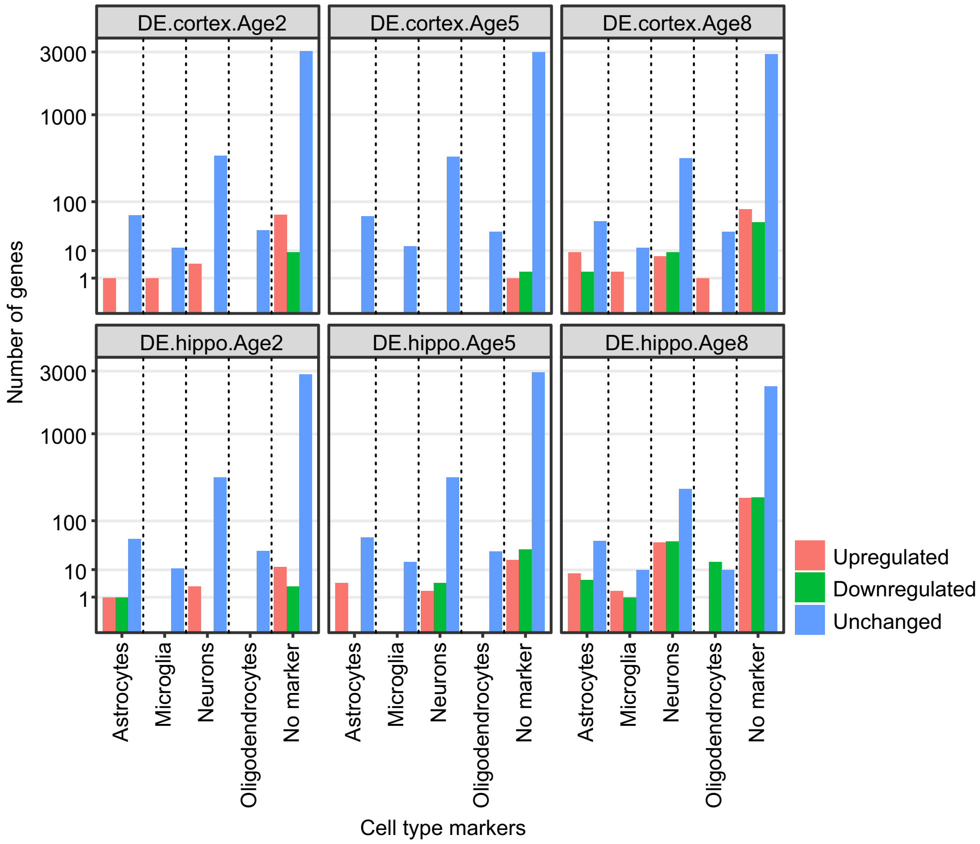

Figure S5. Enrichment analysis of cell-type-specific marker proteins among dysregulated proteins in brains of 5xFAD mice.
The numbers of differentially expressed proteins (defined with a pairwise model;y-axis) that are unchanged (blue), down- (green) or up-regulated (red) and enriched in specific cell types (x-axis) are shown. Analyzes are based on mean values of measured intensities from five biological replicates of tg mice per age and tissue (n=5).

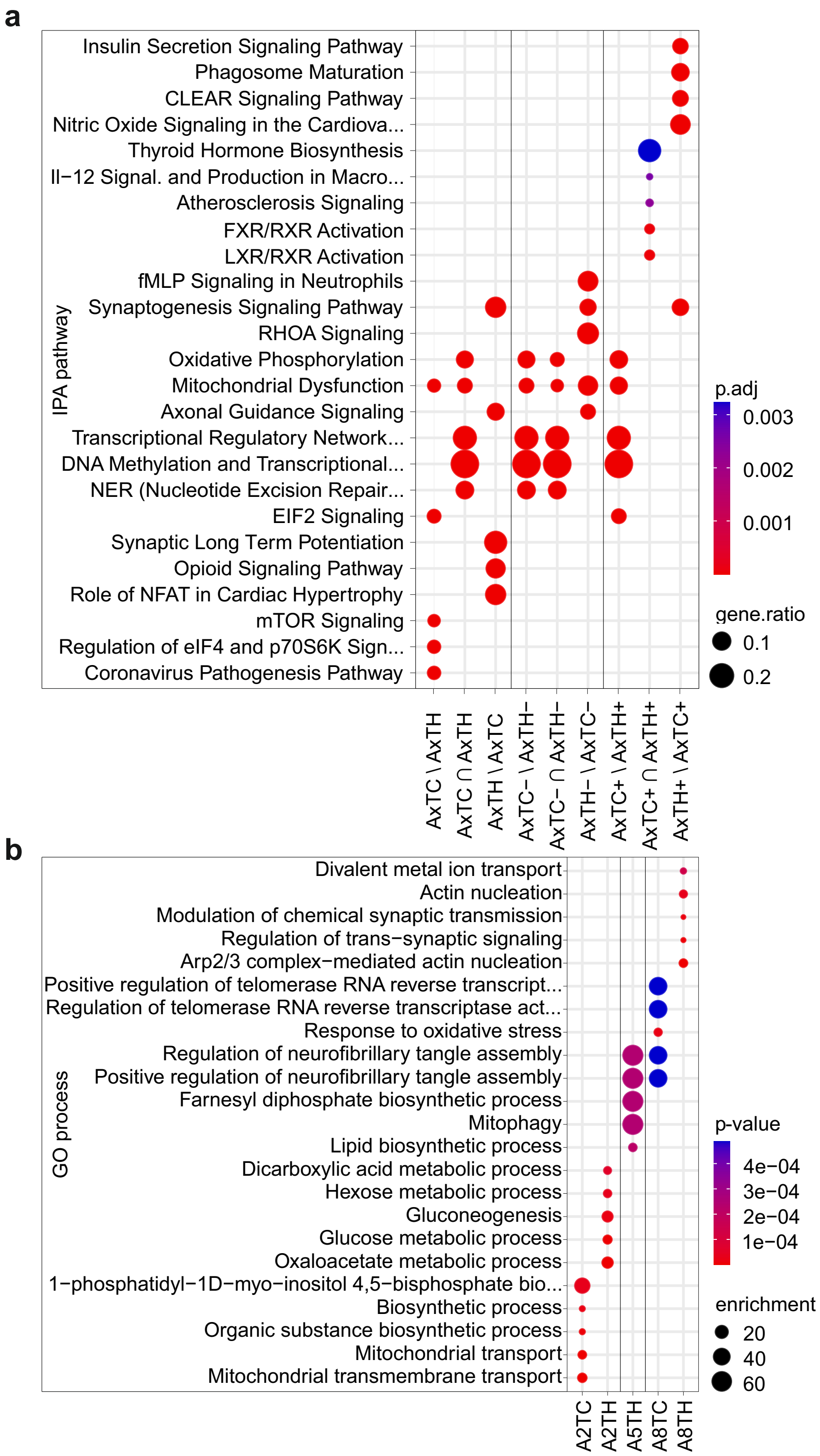

Figure S6. IPA and gene ontology enrichment analyses of differentially expressed proteins (DEPs) defined with the full model in cortical and hippocampal tissues of 5xFAD mice
(a) Ingenuity pathway analysis (IPA) to assess altered cellular processes of dysregulated proteins in hippocampal and cortical tissues; groups of dysregulated (left), downregulated ("-", middle) and upregulated proteins ("+", right) across all ages (2, 5 and 8 months) were analysed considering differences ("\") and intersections ("∩") of proteins. The identifiers were denoted analogously as in Figures 2a and 2d-f. The statistical significance of the association between the DEPs and the canonical pathway proteins was measured with a right-tailed Fisher's exact test to calculate the p-values, adjusted by the Benjamini-Hochberg multiple testing correction. **(b)** Gene ontology (GO) enrichment analysis of the DEPs identified in hippocampal and cortical tissues of 2-, 5- and 8-month-old 5xFAD tg mice. The enrichment was defined as described in the methods section. Statistical significance was determined by computing an exact p-value of a given minimum hypergeometric (mHG) score, corrected for multiple testing. All analyzes are based on mean values of measured intensities from five biological replicates of tg mice per age and tissue (n=5).

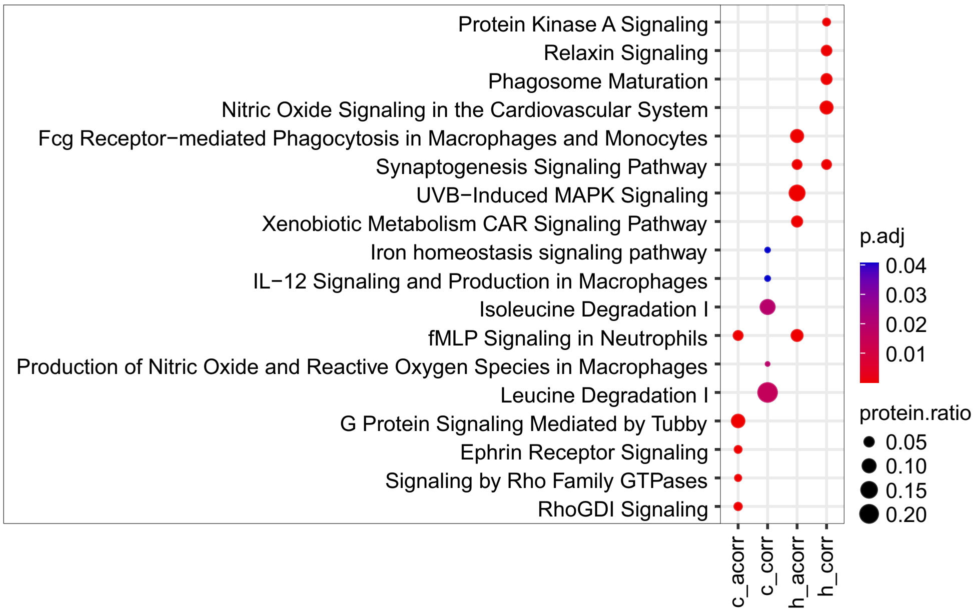

Figure S7. Ingenuity pathway analysis (IPA) of Aβ-correlated and anticorrelated DEPs defined by the pairwise model in brains of 5xFAD mice.

Ingenuity pathway analysis with Aβ-correlated and anticorrelated DEPs identified in hippocampal and cortical tissues. The statistical significance of the association between the DEPs and the canonical pathway proteins was measured with a right-tailed Fisher's exact test to calculate the p-values, adjusted by the Benjamini-Hochberg multiple testing correction. All analyzes are based on mean values of measured intensities from five biological replicates of tg mice per age and tissue (n=5).

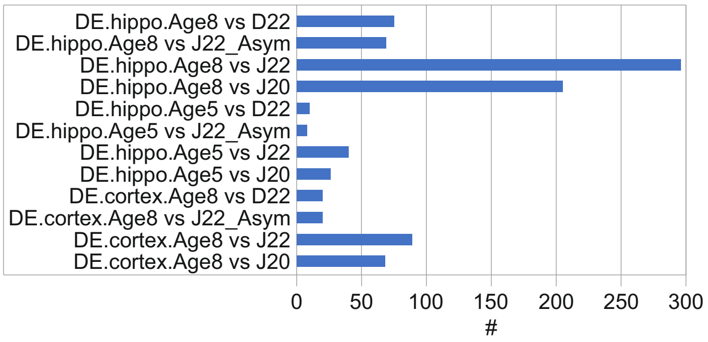

Figure S8. Numbers of pairwise common DEPs in the mouse datasets (DE.cortex.Age8, DE.hippo.Age5, DE.hippo.Age8) and datasets from human studies (J20, J22, J22_Asym, D22).

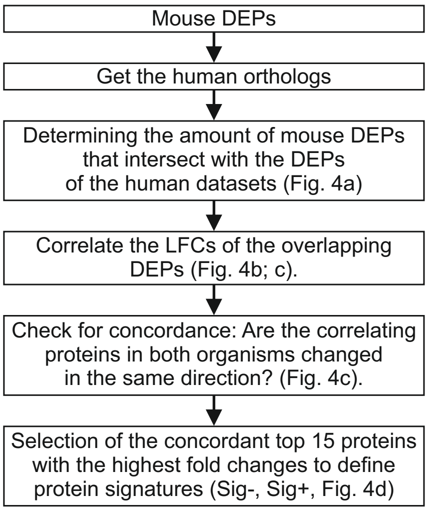

Figure S9. Strategy to define mouse protein signatures that are concordantly altered also in AD patient brains. Schematic representation of the strategy to generate potentially patient-relevant mouse protein signatures (Sig- and Sig+).

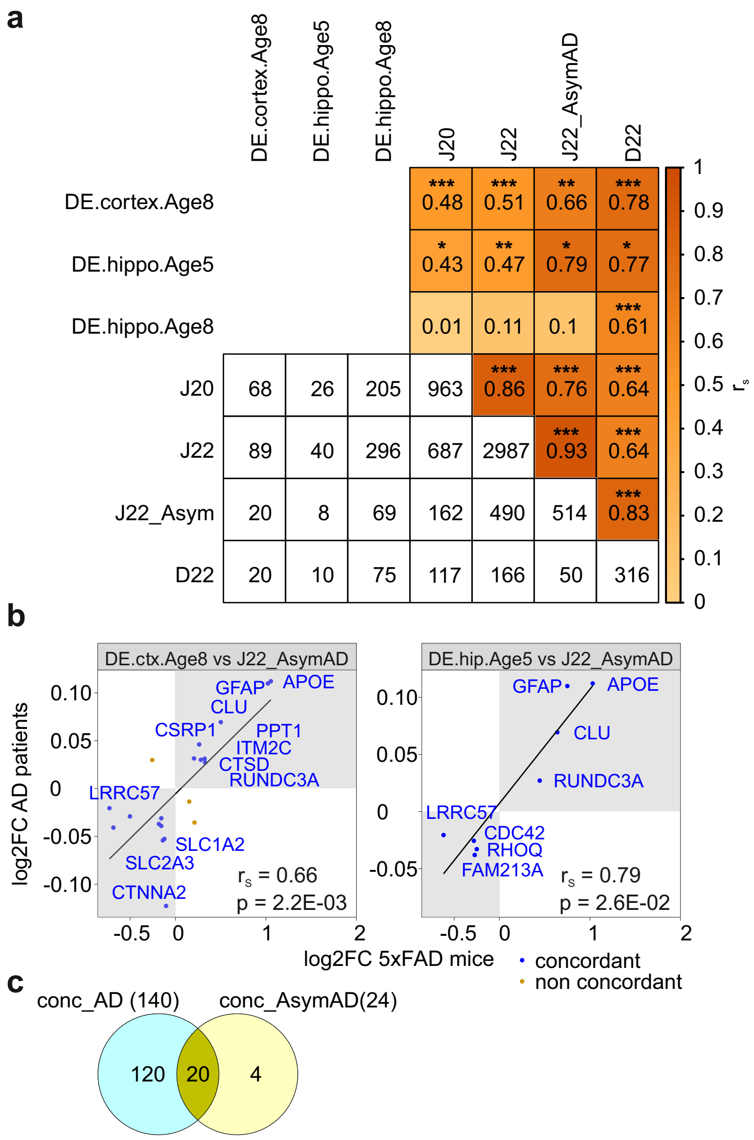

Figure S10. Investigation of the overlap of DEPs in brains of 5xFAD mice with DEPs in brains of asymptomatic AD (AsymAD) patients.

(a) Correlation analyses were performed to investigate the protein abundance changes detected in cortical and hippocampal tissues of 5- or 8-month-old 5xFAD mice (DE.hippo.Age5, DE.hippo.Age8 and DE.cortex.Age8) and human AsymAD patient data from Johnson 2022. To demonstrate the similarity, the correlations shown in Figure 4 with human AD datasets obtained from Johnson 2020 (J20, [40]), Johnson 2022 (J22, [41]), and Drummond 2022 (D22, [39]) are again included here. The colored half of the plot indicates the degree of correlation assessed by Spearman correlation coefficients (r_s), while the corresponding FDR-adjusted p-values are shown above these metrics (*, $p<0.05$; **, $p<0.01$; ***, $p<0.001$). The diagonal line represents the total number of DEPs in the human datasets. The lower left half of the graph depicts the number of overlapping dysregulated proteins in each pair of the investigated datasets. **(b)** Example Spearman correlations and concordance representations of dysregulated proteins from 5xFAD mice (DE.ctx.Age8 and DE.hip.Age5) versus human AsymAD patient data from Johnson 2022 are presented. The datasets are denoted as in Figures 2a and 4a. Proteins concordantly up- or downregulated in 5xFAD mice and human AD patient brains are shown in grey quadrants and marked in blue. Proteins of interest with highly significant fold changes are indicated with gene names. Correlating but non-concordant proteins are marked in brown. **(c)** Investigation of the overlap of DEPs concordantly up- or downregulated in brains of 5xFAD mice, postmortem AD and AsymAD patients. The conc_AD dataset includes all DEPs that are concordantly up- or downregulated in the hippocampus or cortex of 5xFAD mice and AD patients, while conc_AsymAD includes proteins that are concordantly up- or downregulated in the hippocampus or cortex of 5xFAD mice and AsymAD patients. The total numbers of DEPs in each dataset are indicated in brackets. The human datasets were obtained from Drummond 2022 (D22, [39]), Johnson 2020 (J20, [40]), and Johnson 2022 (J22, [41]).

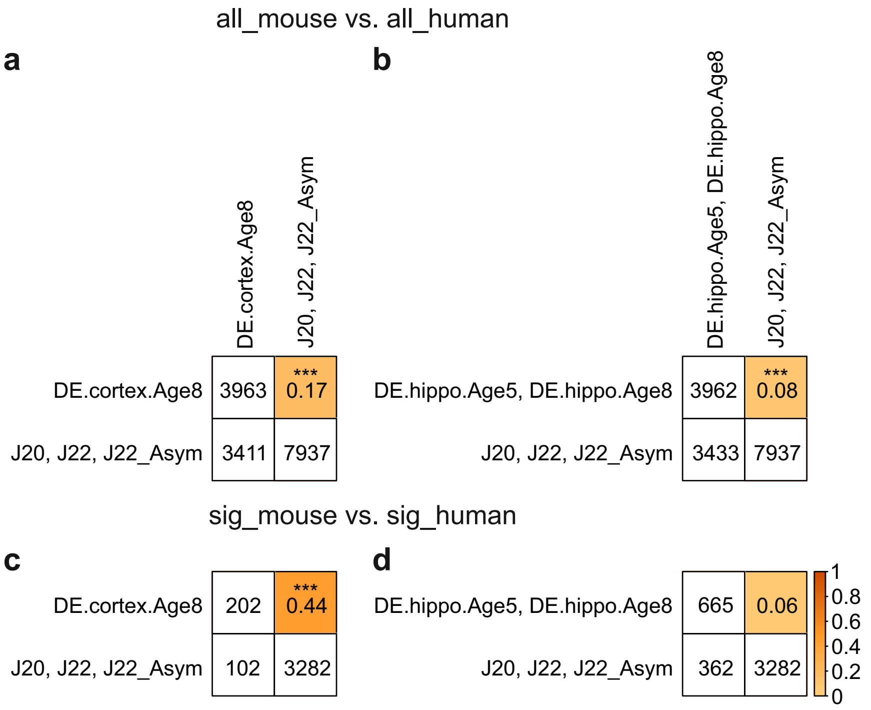

Figure S11. Analysis of the correlation of protein effect sizes between 5xFAD mouse and AD patient brains for proteins present in all studies. To study protein abundance changes in cortical and hippocampal tissues of 5- or 8-month-old 5xFAD mice (DE.hippo.Age5, DE.hippo.Age8, and DE.cortex.Age8) and human patient data from Johnson 2020 and Johnson 2022, correlation analyses were conducted. The protein sets from the hippocampal mouse datasets (DE.hippo.Age5, DE.hippo.Age8) and cortical human datasets (J20, J22, J22_Asym) were merged separately. The correlation analysis included proteins that were detected in either **a)** or **c)** the mouse cortex (at age 8) or **b)** or **d)** the hippocampus (at age 5 and 8), and in all human datasets. The correlations of all proteins (**a)** and **b)**) as well as the correlation of significantly altered proteins (**c)** and **d)**) are presented. The plots depict the degree of correlation, measured by Spearman correlation coefficients (r_S), with the colored square indicating the strength of the correlation, and the corresponding FDR-adjusted p-values (*, p<0.05; **, p<0.01; ***, p<0.001). The square diagonal to the colored square indicates the number of overlapping dysregulated proteins in the pair of investigated datasets. The top-left and bottom-right squares display the total number of DEPs in their respective datasets.

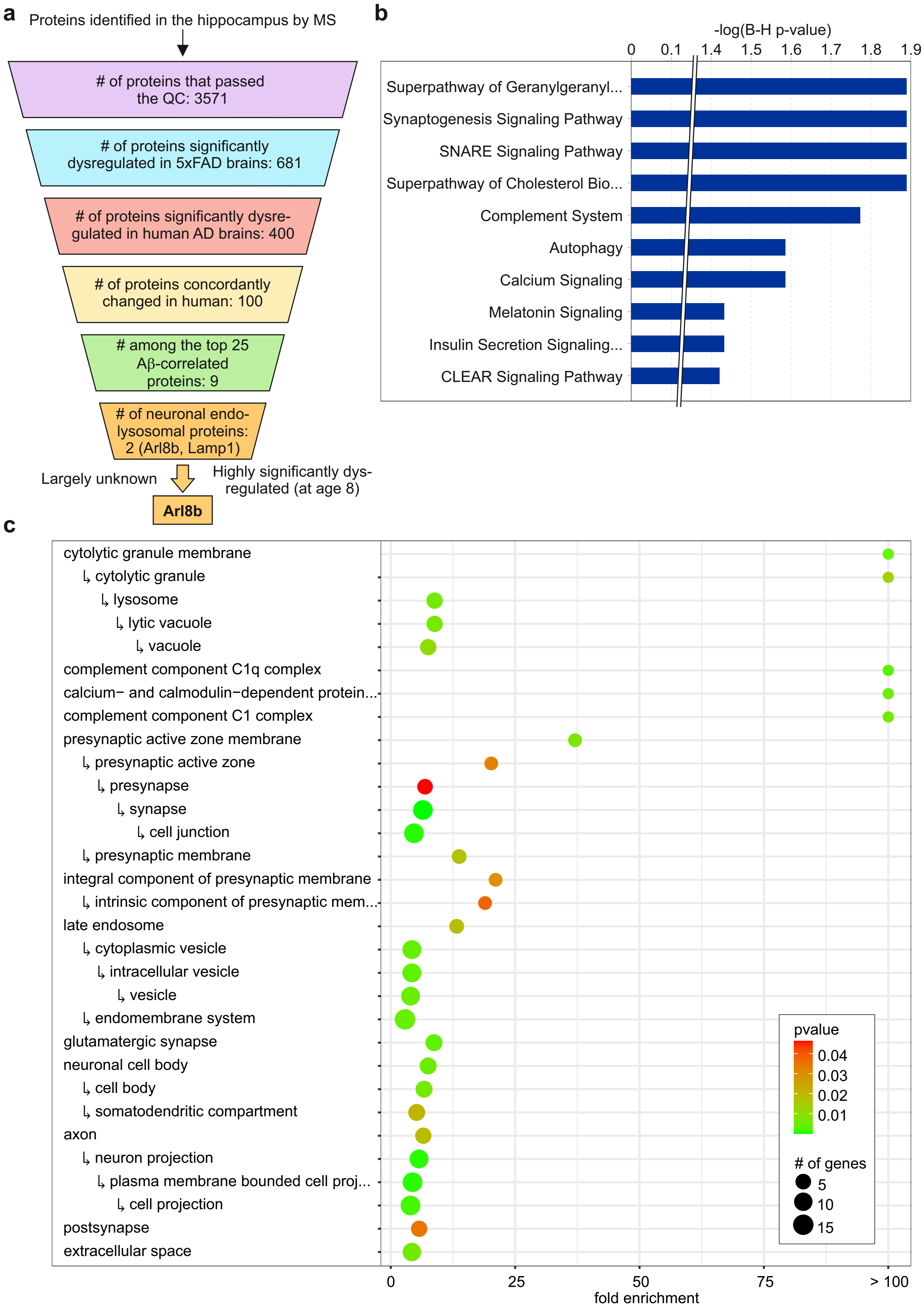

Figure S12. Selection of the neuronal lysosome-associated protein Arl8b by step-by-step data filtering and functional analysis of the top 25 Aβ correlating proteins.
a) Arl8b was selected based on a rigorous selection process. The criteria for the selection of Arl8b from quantitative mouse proteomics data are described in detail in the methods section. **b)** IPA pathway enrichment analysis of the top 25 Aβ correlating proteins in the hippocampus. All statistically significant enriched IPA pathways (FDR corrected pvalue < 0.05) are shown. The x-axis displays the negative logarithm of the FDR corrected pvalue. **c)** GO term enrichment analysis of the top 25 Aβ correlating proteins in the hippocampus. All statistically significant GO cellular components (FDR corrected pvalue < 0.05) are shown as a tree structure. The x-axis reflects the fold enrichment of the genes observed under the top 25 Aβ correlating proteins over the expected ones, that relate to the entire mouse genome. If the fold enrichment is greater than 1, it indicates that the GO category is overrepresented. The FDR corrected pvalues are represented by colors, the numbers of genes mapping to an annotation data category are denoted by the bubble size.

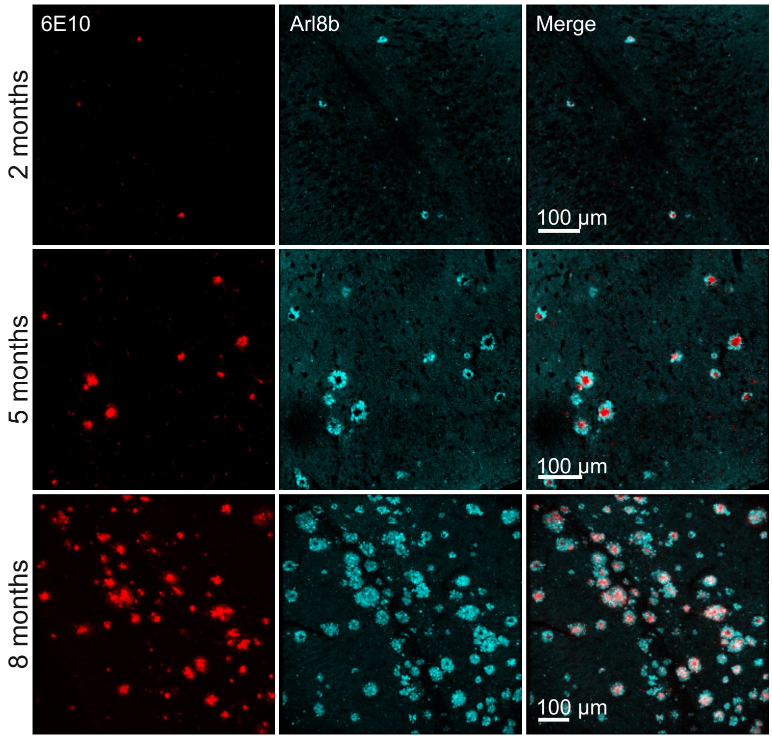

Figure S13. Immunofluorescence analysis of 5xFAD brain slices.
Slices of 2-, 5- and 8-month-old mice were stained with AlexaFluor594-labelled 6E10 antibody (red) and anti-Arl8b antibody detected with AlexaFluor647-labelled anti-rabbit IgG (turquoise).

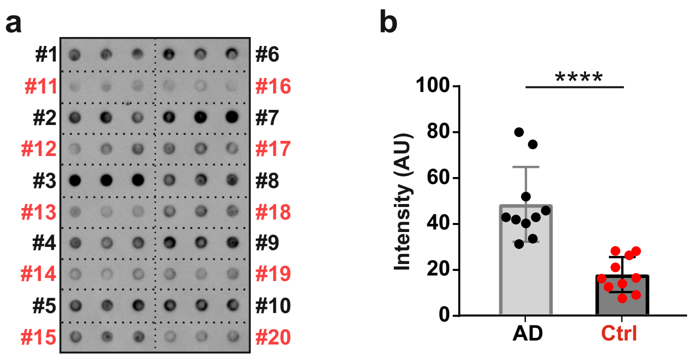

Figure S14. Analysis of Arl8b protein aggregates using human brain homogenates derived from AD patients and control individuals.
(a) Detection of Arl8b protein aggregates in postmortem brain homogenates of 10 AD patients (1 to 10, black lettering) and 10 age-matched controls (11 to 20, red lettering) using a native MFA. Triplicates per sample were filtered. For immunodetection of Arl8b protein an anti-Arl8b antibody was used. **(b)** Quantification of protein retained on filter membranes was performed using an Aida image analysis software. The statistical significance was assessed with an unpaired, two-tailed t test (****, $p < 0.0001$).
